# Sex as a Biological Variable in Research: the New Policy of Balkan Medical Journal

**DOI:** 10.4274/balkanmedj.2018.5.0001

**Published:** 2018-09-21

**Authors:** Zafer Koçak

**Affiliations:** 1 Department of Radiation Oncology, Trakya University School of Medicine, Edirne, Turkey

About 15 years ago, when Professor Dr. Lawrence B. Marks from Duke University wanted me to investigate the difference in toxicity of thoracic radiation between males and females, I first noticed the importance of sex difference in scientific research. Not surprisingly, I was also unfamiliar with the terminological distinction between sex and gender. In the literature search at that time, there were very little data on sex-specific differences in the radiation toxicity of normal tissues. I had noticed that several researchers chose to ignore sex as a variable. Since then, it is discouraging to see that only very few studies on sex-specific differences in radiation sensitivity have been added to the literature (1,2).

I have to admit that the European Association of Science Editors (EASE) conference in Bucharest once again reminded me of the importance of sex and gender equity in research.

It is not unfair to say that the awareness of the editors and researchers of this subject from Turkey is low. Therefore, it is also clear that the editors of Turkish journals need an organization like the EASE.

As I became more familiar with sex and gender issues, I realized that the first steps were already taken before the 2000s (3). In 1993, the National Institutes of Health (NIH) launched a policy to ensure the inclusion of women in NIH-funded clinical research. Later, other funding agencies, including the Canadian Institutes for Health Research, the World Health Organization, and the European Commission, explicitly requested sex and gender analysis for health research (3). Other efforts in this regard include the guidelines developed by the International Committee of Medical Journal Editors (ICMJE) and the EASE. The Sex and Gender Equity in Research (SAGER) guidelines were developed by the Gender Policy Committee of the EASE for reporting sex and gender in all types of science publications (4,5).

With those recent efforts, the number of women participating in clinical trials has increased in the last two decades; however, women are still inadequately represented in studies. Even if women are involved in clinical trials, the data are often not analyzed separately by sex. Single-sex study design is not only incomplete but may also be harmful, as reported by Heinrich J in 2001 (6). In her letter, she explained that 10 drugs were withdrawn from the US market due to life-threatening health effects between 1997 and 2000; eight of them constituted more health risks for women. Because in the analysis of the studies, sex or gender was not a biological variable.

This path, opened by the funding institutes and organizations, was a guide for the journal editors. Journal editors started to set rules that encourage authors to consider the analysis and reporting of sex and gender differences. Thus, several peer-reviewed journals have recently adopted sex and gender reporting policies (7).

Balkan Medical Journal has successfully implemented the ICMJE recommendations such as ethical approval requirements, conflicts of interest and financial statements, authorship, and clinical study registry requirements, but it does not have a specific policy on sex and gender reporting.

In the article by Clayton JA (8), I read some suggestions on sex as a biological variable for searching the literature and planning the research. I found it very helpful, and I thought that these suggestions should be represented in a table to get more attention from the readers and authors (Table 1).

We, the editorial team of Balkan Medical Journal, are happy to announce the new policy (sex and gender reporting) for Balkan Medical Journal. As a result of this accepted policy, we decided to follow the SAGER guidelines (Table 2) (5). Authors are strongly recommended to use the terms sex (for reporting biological factors) and gender (for reporting identity, psychosocial, or cultural factors) correctly. We encourage all authors to analyze and report sex and gender differences in their research, wherever appropriate.

Editors of Balkan Medical Journal are thrilled to be a part of the global efforts to improve the reporting and evaluation of sex and gender differences in scientific research. We hope that our editors, reviewers, and authors will quickly adopt this guide and contribute to a better science and health outcome.

## Figures and Tables

**Table 1:**
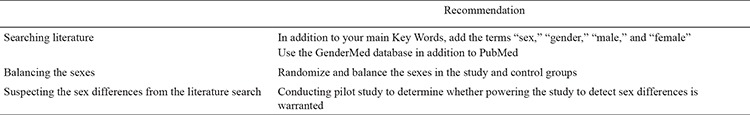
Clayton’s suggestions for planning research^8^

**Table 2:**
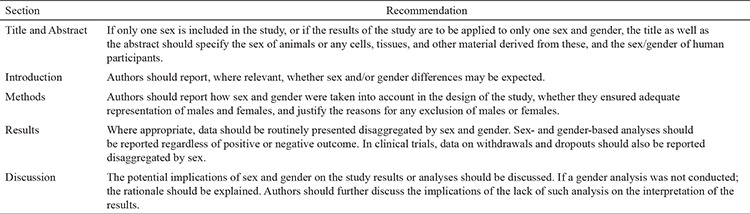
Sex and Gender Equity in Research (SAGER) recommendations^5^
